# Loss of Hair

**Published:** 1880-01-20

**Authors:** John V. Shoemaker


### *LOSS OF HAIR.*

BY JOHN V. SHOEMAKER, A.M., M.D.

Hair is merely modified epithelial tissue enclosed in follicles, and is found over almost the whole surface of the skin. It may be long, as on the scalp, short and thick, as on the eye-lashes, or soft and delicate, as over the greater part of the surface of the body. The length of the hair ranges from one line to one yard, and varied much in the two sexes and in the different parts of the body. According to the accurate observation of Wilson, the thickness of the hair ranges between 1–1500 to 1–400 of an inch, being finer in children than in adults, and coarser in women than in men.

As regards color, the hair may be either black, gray, white, light brown, dark brown, red, chestnut, or flaxen. The hue will be modified according to the quantity of pigment present and the manner in which it is arranged in the fibrous part of the hair. The hair is lightest in Northern climates and becomes darker as we pass southward. The hair acts as protection in both cold and warm climates. It is of service in assisting to maintain the temperature of certain parts of the body, and assists in eliminating some of the effete matters from the system. It is a means of defense for the eyes and the nostrils, and in certain occupations prevents the entrance of dust into the lungs. It likewise serves to adorn and beautify the features. Its loss, which is of frequent occurrence in young persons and in those in the middle period of life, is therefore, not only a source of great inconvenience and distress, but also of great disfigurement.

This important part of the economy seldom receives proper attention from either physician or patient. Patients should be made aware of the evil resulting from neglecting the hair, and physicians should understand thoroughly how to prevent its loss; and, when such loss occurs, the proper manner of treating it and avoiding disfigurement should be known.

The hair may fall out in patches or by general thinning, and it is to this latter class of cases that I shall briefly allude. The shedding of the hair by the atrophy of the tissue with the advance of age is preceded by general thinning, but as it is a natural change it should not be regarded as a disease, and will not, therefore, require any particular attention. Loss of hair, as a result of diseased action occurs in both sexes, but more frequently in males. It may occur at any period of life, but is most common in young adults. It may concern the entire surface, or only a portion. It usually, however, involves the scalp or the beard. When the disorder involves the head it usually begins about the vertex and extends until all the superior and anterior part of the head is bereft of hair. The hair may be shed rapidly, coming out especially during the act of combing or brushing; or it may fall out slowly and progressively, and this is the course in the majority of cases. In one variety of the disease, the scalp is to all appearances healthy, no crusts or scales being present. The hairs are dull, wanting in pigment, and when compared with the healthy hairs on the side and back of the head are found to have decreased in both length and thickness. These hairs are cast off from time to time, and are replaced by finer ones until the entire superior and anterior portions of the scalp are filled with very short and fine hairs. The disorder speedily progresses for years without attracting the patient's attention until the loss is pointed out by some friend or acquaintance.

If the diseased condition is not arrested, the small downy hairs will in turn be cast off until the crown of the head becomes permanently bald. In these cases the hair over the rest of the body usually goes on growing in the natural condition. The patient, during the period of the shedding of the hair, will appear to be in good health, but a careful examination into the case will reveal the evident cause of this disorder. It will be found, as has been suggested by Von Barensprung, that this variety of the disease is due to a failure in the nerve power of the part. It occurs among those who are over-worked, either mentally or physically. It is most liable to result from excessive mental strain, especially among professional men and men of active business duties. It is observed to result from unsuitable food, debilitating diseases, anxiety, grief, fast living, heat, neuralgia and rheumatism.

The next variety of general thinning of the hair occurs also upon the crown of the head. The scalp is covered with dry scales or crusts of various dimensions, or both may be present in the same case. Excessive cell proliferation takes place all along the course of the follicles. The small dry and white scales are loosely distributed over the different portions of the scalp. When the surface is closely examined with a magnifying glass, the scales are found to plug up all the open follicles. If this scurfy material is detached the surface of the scalp will present a gray and atrophied appearance. The hair is dry, withered, and lacks lustre. It is detached slowly and successively in combing, and even during the ordinary movements, until it becomes very thin or permanent baldness occurs. The crusts when present are firmly attached in the follicles, and when removed will expose a red and unhealthy condition of the part. If the scalp is not properly cleansed in either case, the accumulation of discharged sebum will alter and obliterate the follicles. This abnormal condition of the part will sometimes cause itching, and the scratching thus induced may cause eczema. This secondary change may mat the hair and crusts together and quickly kill the growth of the hair.

Those who are afflicted with this variety of the disease will generally have a lowered vitality, which will be expressed in cold extremities, dry and pale skin, or in some functional derangement. Syphilitic poison may give rise to an infiltration, or ulceration around the sebaceous glands and follicles, and so result in the loss of the hair. Ringworm in children will often lead to destruction of the follicles and end in baldness of the affected part. Among other prominent causes are erysipelas, variola, lupus psoriasis, dyeing the hair, long-continued pressure on the scalp, and wearing coverings on the head that cause profuse sweating. Although the scalp is more abundantly supplied with hair in women than in men, owing to the hair being more scanty over the rest of the body and the superabundance of nutritive pabulum being carried to the head, yet the majority of women prevent the hair from growing in the proper manner. It is the incessant curling, crimping, and the injurious modes of dressing the hair that destroy its beautiful, soft and glossy appearance, and arrest its growth. It is also the bleaching and the powdering of the hair that render it dull, plug up the follicles and cause it to be cast off in handfuls.

If the hair received the proper attention, many cases of general thinning and premature baldness might be prevented. The use of close-fitting hats and bonnets and the tension of the hair should be avoided. It should be exposed to the air every day as this will assist in nourishing it. It should likewise be regularly cleansed by bathing with soap and water, as with the rest of the body, so as to wash off the effete material that exudes from the surface. It is impossible to restore the hair lost by the natural changes of advanced years, or when the follicles are destroyed, or cicatrized; but if the follicles with their papillæ are healthy and no atrophy of the tissue has taken place, partial or complete restoration of the hair is possible, providing the remedies are properly employed. The majority of cases will require both internal and external treatment. Each case will demand special management, according to the causes of the disorder. For instance, syphilitic taints will require specifics; rheumatic and neuralgic conditions will call for anti-rheumatic and neuralgic remedies; unsuitable nutrition will demand good, substantial food; and crimping, powdering, bleaching and dyeing the hair will require an abandonment of the injurious habit.

With regard to those cases in which the patient is apparently in good health and is unconsciously losing the hair, tincture of ignatia, mercury, or sulphurous acid, have, in my experience, been the most successful remedies. Some cases respond nicely to ten drop doses of tincture of ignatia, or one half a drachm of sulphurous acid three times daily, with bitter tonics, while others show a more decided improvement upon very minute doses of mercury. Mercury, when given in very small doses, is a very decided tonic, and is especially valuable in the first variety of the disease as described above. The failure of the constitutional treatment to act promptly in many cases is owing to the manner of employing the medicine, and not to the use of any special remedy. For example, should mercury be given in the ordinary dose, or should it be persisted in for a long time, it will be found to pass off by the secretions and do very little towards benefitting the patient. If, on the contrary, mercury be administered for a short period in the amount of 1–15 to the 1–20 of a grain, of either the mild or corrosive chloride, and then the tincture of ignatia with a bitter tonic be substituted, and so vary the remedies from time to time, according to the patient's condition, the result will be either beneficial or successful.

The internal treatment should always be aided by appropriate external applications. In the first place, the head should be immersed in cold water every night and afterwards rubbed with a rough towel, until a warm glow is produced over the surface of the scalp. The head should likewise be thoroughly brushed from time to time until redness appears; and this brushing is especially serviceable in women. Active friction to the scalp, in order to awaken the sluggish circulation, is indispensable, and the friction should be increased or diminished according to the insensibility or sensibility of the part. The short, ragged and lusterless hairs should be cut off at the ends, or plucked out. This will strengthen the hair and add tone and vigor to the scalp. It is also decidedly the best plan, when the long healthy and normal hair has disappeared and the surface is covered with a fine, downy growth, to shave the scalp. This shaving should be repeated about every three or four weeks for some time, and should upon each occasion be succeeded by rubbing the skin effectually until every part is thoroughly stimulated. In addition, occasionally passing a gentle current of electricity over the head will add very much in promoting the growth of the hair. It is of equal importance in the first variety to use some mild stimulating remedy that may assist materially the preceding practice. After washing the head I have used with success in these cases a lotion made as follows:

℞ Strong water of ammonia.............................. 1½ ounce
Spirits of rosemary and alcohol................. 1 ounce of each
Tincture of capsicum.............................. 2 drachms.
Tincture of nux vomica................................ 1 drachm.
Spts. chloroform.......................................... ʒj.

This solution should be applied with a sponge to the scalp three or four times a week. It should be rubbed in effectually until a warm glow is produced, and then the head should be bathed with warm water.

In the second variety, when the head is covered with the well-known dandruff, I usually wash up the scalp with medicated soap and water every two or three days. The soap that I prefer to use in such cases has been made at my suggestion by Mr. L. Wolf, of this city. It is composed of one and a half ounces each of oil of theobroma and olive oil; two drachms of German chamomile flowers; one drachm of precipitated sulphur, and one ounce of a weak solution of caustic soda.

This soap removes all the dandruff from the head and produces a gentle stimulant and astringent effect upon the follicles and glands of the scalp. It will be necessary, if the thinning or the loss of the hair is due to the formation of crusts, to remove them by oil dressing or poultices. The scalp can be saturated with oil, covered with an old piece of flannel and oil silk, and in from twelve to twenty-four hours the masses will be sufficiently soft so as to be easily removed. The parts can then be washed with the medicated soap and sponged from time to time with the lotion previously mentioned. All that remains to be done locally, after stimulation in the first case, and the removal of the crusts and scales in the second, is to further induce the growth of the hair by the use of suitable liniments or pomades. I always prefer the use of oily preparations in place of cosmetic or pomades, as the latter are very liable to become rancid and do more harm than good. I have employed with advantage, to lubricate and induce the growth of the hair, the following liniment:

| | | |
|---|---|---|
| ℞ | Tannate of glycerine........................... | 2 ounces. |
| | Tincture of cantharides........................... | 1 drachm. |
| | Tincture of nux vomica........................... | 1 drachm. |
| | Oil of erigeron........................... | 2 drachms. |
| | Olive oil........................... | 1½ ounce. |
| | Oil of rose........................... | 5 drops. |
| | Oil of verbena........................... | 5 drops. |

This should be applied once every day until all the dull and lustreless hairs have disappeared and there is some evidence of returning nutrition in the scale and hair.—*Med. Bulletin.*

